# Comparative Analysis of Untargeted Metabolomics in Tolerant and Sensitive Genotypes of Common Bean (*Phaseolus vulgaris* L.) Seeds Exposed to Terminal Drought Stress

**DOI:** 10.3390/metabo12100944

**Published:** 2022-10-05

**Authors:** Mayavan Subramani, Carlos A. Urrea, Venu Kalavacharla

**Affiliations:** 1 Molecular Genetics and Epigenomics Laboratory, College of Agriculture, Science and Technology, Delaware State University, Dover, DE 19901, USA; 2 Panhandle Research and Extension Center, University of Nebraska, 4502 Avenue I, Scottsbluff, NE 69361, USA

**Keywords:** metabolites, common bean, genotypes, seeds, terminal drought stress

## Abstract

Many environmental stresses can affect the accumulation of metabolites in plants, including drought. In the present study, we found a great deal of variability in the seed metabolic profiles of the tolerant (Matterhorn, SB-DT2 and SB-DT3) common bean genotypes in comparison to the sensitive genotypes (Sawtooth, Merlot and Stampede) using ultrahigh performance liquid chromatography−tandem mass spectrometry (UPLC-MS). The genotypes were grown in the field and subjected to drought stress after flowering (terminal drought stress). We aimed to investigate the accumulation of genotype-specific metabolites and related pathways under terminal drought stress by comparing tolerant and sensitive genotypes within a race. A total of 26 potential metabolites were identified across genotype comparisons. Significant metabolic pathways, including monobactam biosynthesis, flavone and flavonol biosynthesis, pentose phosphate pathway, C5-branched dibasic acid metabolism, cysteine and methionine metabolism, vitamin B6 metabolism and flavonoid biosynthesis, were derived from the enriched metabolites. Many of these metabolic pathways were specific and varied with genotype comparisons. SB-DT2 vs. stampede revealed more significant metabolites and metabolic pathways compared to Matterhorn vs. Sawtooth and SB-DT3 vs. Merlot under terminal drought stress. Our study provides useful information regarding the metabolite profiles of seeds and their related pathways in comparisons of tolerant and sensitive common bean genotypes under terminal drought conditions. Further research, including transcriptomic and proteomic analyses, may contribute to a better understanding of molecular mechanisms and nutritional differences among seeds of common bean genotypes grown under terminal drought conditions.

## 1. Introduction

Metabolites are essential to plant metabolism and impact all biological processes viz., survival, growth, development, plant defense and response to biotic and abiotic stresses. These low-molecular-weight compounds (<1500 Da) of primary and secondary metabolites govern the functional status of plants, which includes plant interaction with their environment. It has been previously shown that untargeted profiling of metabolites will contribute to a better understanding of strategies for crop improvement, as metabolites link the genotype with the phenotype [1,2,3].

Plants have been known to undergo essential metabolic reprogramming to synthesize metabolites to adapt to stressful conditions. Several metabolic components of plants are affected by abiotic stress, and most of these are in plants’ primary and secondary metabolic pathways. These compounds allow plants to adapt and survive in unfavorable conditions [4,5]. Metabolic profile changes in response to biotic and abiotic stress have been reported [6,7]. Additionally, metabolites have often been used in crop breeding as selection biomarkers [8,9].

Metabolic responses are specific to the type of stress the organisms undergo. Accumulating specific metabolites, their related pathway, and their metabolic network depends on the type of stress. Commonly occurring metabolites under different stresses also vary, depending on the stress conditions and accumulation time of these metabolites. Earlier studies have reported that drought stress changed the accumulation of metabolites in peanuts, with sugar, sugar alcohol, organic acids, and fatty acids being the primary metabolites altered [10]. These compounds accumulated specifically to protect against oxidative damage to cellular components [2]. Thus, the metabolites can be potentially used as a good candidate to reveal the effect of specific stress [11]. Additionally, plant genotypes influence the production of metabolites in response to stress conditions. Production of phenolic compounds differed among the peanut genotypes in response to drought stress [12]. Tolerant and sensitive soybean genotypes showed diverse metabolic responses to drought stress [13]. In the wheat genotypes, differences were observed in the levels of metabolites accumulated in response to drought [14].

Common bean (*Phaseolus vulgaris* L.) is an important food legume consumed worldwide for its nutrition-rich seeds and pods [15,16]. About 47 to 69% of common bean yield is affected by drought stress. Most bean production is affected by intermittent and terminal droughts, which affect pod development and seed production [17,18,19]. Drought affects seed metabolites in several ways, including reallocation of metabolites, formation of new metabolites, and a reduction in their content [20,21,22]. Therefore, comprehensive seed metabolomic profiling is still required to extend the knowledge of metabolite variance in response to changing environmental conditions [1].

Metabolites that are formed in response to abiotic stress also possess pharmacological effects. Several phenolics and flavonoids produced in response to abiotic stresses possess high antioxidative characteristics [23]. Polyphenol compounds present in the seed coat of the common bean have demonstrated significant antioxidant activity and protective effects against oxidative stress [24]. Moderate drought stress increased the levels of phenolic acids in the leaves of safflower (*Carthamus tinctorius* L.) [25]. *Achillea* species exhibited higher phenolic acids and flavonoids under severe drought conditions [26]. We believe that utilizing untargeted metabolomics in the present study will enable us to identify bioactive and drought stress-responsive metabolites [23].

The purpose of this study was to understand genotype-specific metabolite accumulation and its associated metabolic pathways in comparing the tolerant genotypes to the sensitive genotypes of common bean seeds within a race under terminal drought stress. In our comparison of genotypes, we found a great deal of variation in seed metabolic profiles. The genotype comparison of SB-DT2 vs. Stampede revealed higher significant metabolites and their related pathways than the other genotype comparisons. Possibly, our study will provide new insights into the seed metabolic characteristics of common bean genotypes grown under terminal drought stress.

## 2. Materials and Methods

### 2.1. Experimental Design

Six genotypes of common bean (*Phaseolus vulgaris* L.) (Table S1) were grown in a field with silt loam soil (Typic Ustorthents) (41°56.6′ N, 103°41.9′ W, 1240 m elevation). The soil consisted of 75% silt, 15% sand, and 10% clay. It had a cation exchange capacity of 17 meq/100 g, a pH of 7.8, and an organic matter content of 14 mg/g. There was 20.5 kg of residual nitrogen in the field, and 25.9 kg of nitrogen in the manure credit; therefore, no additional nitrogen was applied to the field. To draw comparisons between genotypes, we used the following alphabet. There were three tolerant genotypes: Matterhorn (A1) [27] SB-DT3 (B1), and SB-DT2 (C1) [28], and three sensitive genotypes: Sawtooth (X1) [29], Merlot (Y1) [30], and Stampede (Z1) [31]. The genotypes were compared within the same race (Figure S1, Table S1). The field experiment was conducted according to [28]. Briefly, the soil was prepared uniformly and plowed to promote healthy bean growth. The experiments were conducted in a randomized block design with four replications. Each plot consisted of four 3.6 m rows 56 cm apart. Plants were harvested from the middle two rows (10 feet) of each plot at the end of the drought treatment. The different entries had been randomized with random numbers to avoid any bias. Drought stress was induced after flowering as a terminal drought treatment. At least 50% of the plants reached anthesis (flowering) before a terminal drought was imposed. Until then, drip irrigation was used to irrigate the plants. A total of 135.1 mm of precipitation was recorded during the period prior to flowering. This includes irrigation of the plants twice (101.6 mm) and precipitation of 33.5 mm. There was a total of 16.3 mm of precipitation recorded after flowering, and no irrigation was provided to the plants. A total of 18 samples were collected from 3 sensitive and 3 tolerant genotypes in 3 replicates after 2–3 weeks of drought stress during the R7 stage of development.

### 2.2. Reagents

Acetonitrile, methanol, formic acid, and DL-o-Chlorophenylalanine were purchased from Merck, LC/MS grade (Kenilworth, NJ, USA).

### 2.3. Sample Preparation

The samples were lyophilized to dryness and ground to a fine powder in a 5 mL homogenizer tube at 30 Hz using four 5 mm metal balls on a MM 400 mill mixer. In each tube, 50 mg of the sample was precisely weighed, and then 800 µL of 80% methanol was added. Then, samples were vortexed for 30 s, followed by sonication for 30 min at 4 °C. Next, the samples were kept at −20 °C for 1 h and centrifuged at 12,000 rpm at 4 °C for 15 min. Then, 200 µL of supernatant and 5 µL of DL-o-Chlorophenylalanine (140 µg/mL) were transferred to a vial for LCMS analysis.

### 2.4. Biomolecules Quantification and Agronomic Characters

Lipid and starch contents were determined according to [32] with modifications. Briefly, the seed samples were dried at 105 °C and ground into powder. In total, 1~2 g of samples was extracted with petroleum ether at 65 °C for 1 hr. The samples were dried again at 105 °C and the fat contents were determined. For starch quantification, 0.1 g of powdered samples was extracted with 80% ethanol-H2O to release starch. Then, the samples were subjected to acid hydrolysis (sulfuric acid) in order to release glucose. The glucose concentration was determined using a microplate reader at 620 nm. In addition, agronomic characteristics such as yield, days to flowering and harvest maturity, and 100 seed weight were also measured.

### 2.5. UPLC-MS (Ultraperformance Liquid Chromatography-Tandem Mass Spectrometry)

Separation was performed by Ultimate 3000LC combined with Q Exactive MS (ThermoFisher, Waltham, MA, USA) and screened with ESI-MS. The LC system is comprised of an ACQUITY UPLC HSS T3 column (100 × 2.1 mm × 1.8 μm) with Ultimate 3000LC (Waters Corp, Milford, MA, USA). The mobile phase is composed of solvent A (0.05% formic acid water) and solvent B (acetonitrile) with a gradient elution (0–1 min, 95%A, 1–12 min, 95–5% A, 12–13.5 min, 5% A, 13.5–13.6 min, 5–95% A, 13.6–16 min, 95% A). The flow rate of the mobile phase was 0.3 mL·min^−1^. The column temperature was maintained at 40 °C, and the sample manager temperature was set at 4 °C. Experimental design flow chart (Figure S2).

Mass spectrometry parameters in ESI+ and ESI− mode are listed as follows:

ESI+: Heater Temp 300 °C; Sheath Gas Flow rate, 45 arb; Aux Gas Flow Rate, 15 arb; Sweep Gas
Flow Rate, 1 arb; spray voltage, 3.0 kV; Capillary Temp, 350 °C; S-Lens RF Level, 30%.
ESI−: Heater Temp 300 °C, Sheath Gas Flow rate, 45 arb; Aux Gas Flow Rate, 15arb; Sweep Gas
Flow Rate, 1 arb; spray voltage, 3.2 kV; Capillary Temp, 350 °C; S-Lens RF Level, 60%.

### 2.6. Statistical Analysis

The raw data were acquired and aligned using the Compound Discover (3.0, ThermoFisher Waltham, MA, USA) based on the m/z value and the retention time of the ion signals. Compound discovery software, which uses a variety of plant and animal databases, was used to identify the chemical structures of significant metabolites based on mass and MS/MS fragment data. Additional confirmation was obtained, when necessary, by comparing retention times and MS/MS fragmentation patterns to authentic standards. For multivariate analysis, ions from both ESI− or ESI+ were merged and imported into the SIMCA-P program (version 14.1). As a first step toward visualizing data and identifying outliers, principal components analysis (PCA), an unsupervised method, was used for data visualization and outlier identification. PCA emphasizes variation and highlights patterns in a dataset [33]. Supervised regression modeling was then performed on the extensive dataset using partial least squares discriminant analysis (PLS-DA) or orthogonal partial least squares discriminant analysis (OPLS-DA) to visualize the important metabolites. Then, the significant metabolites were filtered and identified by combining the results of VIP > 1.5 and *p* < 0.05 (*t*-test).

**Figure 1:**
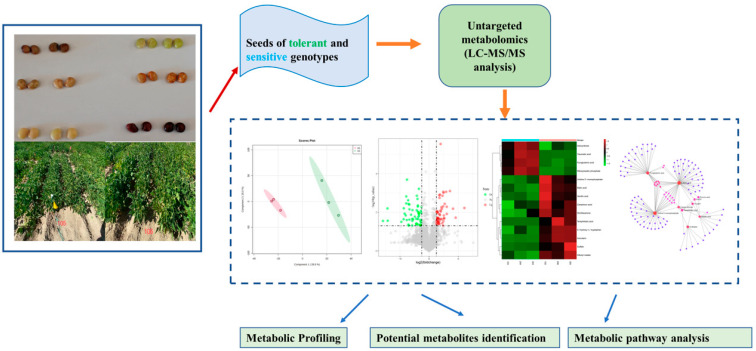
Overview of the untargeted metabolomic profiles of common bean seeds.

Figure 1 illustrates a schematic diagram of the manuscript overview.

## 3. Results

### 3.1. Morphological Traits and Analysis of Starch and Fat Content

SB-DT3 and Sawtooth had a reduction in yield under the drought stress. All genotypes had similar flowering and maturity days (45 to 49 days and 87 to 89 days, respectively) except Matterhorn, which reached maturity at 82 days. All genotypes had a similar 100 seed weight (33 to 35 g) except the Matterhorn genotype, with a low 100 seed weight of 30.8 g, and the Sawtooth genotype, with a high 100 seed weight of 37.3 g (Table S2). Genotype Stampede had a relatively low starch and fat content compared to other genotypes. Merlot and SB-DT2 had a higher starch content than other genotypes, while SB-DT3 and Merlot had a higher percentage of fat content (Figure S3).

### 3.2. Metabolic Profiling and Quality Control

Metabolites were obtained in both negative and positive ionization modes. However, in the negative ionization mode, the total metabolites obtained were higher, 184, whereas, in the positive mode, it was 121 (Table S3). Genotype comparisons are detailed in the Materials and Methods section and Figure S1. The metabolites with a fold change of >2 were greater in the negative ionization mode than in the positive (Table S4). More metabolites were identified in SB-DT2 (C1 vs. Z1) and SB-DT3 (B1 vs. Y1) genotypes with a fold change (FC) greater than 2 compared to other genotypes. Syringic acid, cosmosiin soyasaponin A1, abscisic acid, and ferulic acid were common metabolites observed in both ionization modes across the genotypes (Figure S4). Several metabolites with FC > 3 were identified in Matterhorn genotypes, including ribosylzeatin phosphate, syringic acid, piscidic acid, and jasmonic acid. The SB-DT3 genotype with FC > 3 contained cosmosiin, eriodictyol, mevalonic acid, and vanillin. Several metabolites with FC > 3 were detected in SB-DT2, including cyanidin 3-sambubioside, quercetin 3-arabinoside 7-glucoside, luteolin, xanthine, procyanidin B1, soyasaponin A1, ferulic acid and rutin.

### 3.3. Metabolic Changes in the Genotypes

**Figure 2:**
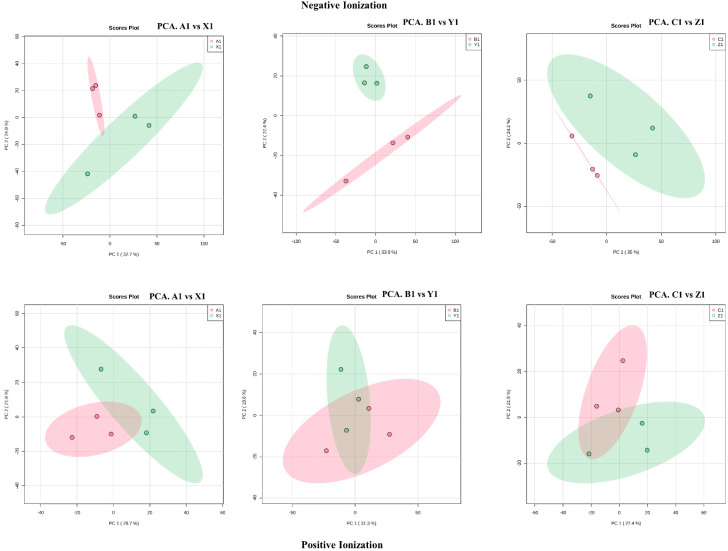
PCA scores plot showing statistically significant unsupervised separation between the metabolites of tolerant and sensitive genotypes.

We used PCA (principal component analysis) to analyze all observations in both ion modes for the metabolite variations. The unsupervised PCA plot in Figure 2 clearly shows the separation between the metabolites with the percentage of variance in the principal components (PC1) and principal components (PC2) for the tolerant and sensitive genotypes. A supervised PLS-DA (partial least squares discriminant analysis) and an OPLS-DA (orthogonal partial least squares discriminant analysis) also showed distinct group separations supporting the PCA analysis (Figure S5). This further allows for a comparison of significantly changed metabolites between two groups.

### 3.4. Variable Importance Projection (VIP)

The VIP values (VIP > 1.5) were used to filter out significantly changed metabolites between the tolerant and sensitive genotypes. There were 79 metabolites in total identified in negative ionization mode and 61 metabolites in positive ionization mode. There were more significantly altered metabolites in the genotypes SB-DT2 (C1 vs. Z1) than in others. Metabolites such as linolenic acid, avicularin, homocysteine, ribosylzeatin phosphate, dibutylmalate, pyroglutamic acid, N-Acetyl-L-glutamate 5-semialdehyde, D-glucuronic acid, linolenic acid, eriodictyol, soyasaponina1, homocysteine, retinoic acid, ophthalmic acid, pyridoxine, d-fructose were detected in both ionization mode across all the genotypes (Table S5).

### 3.5. Volcano Plot

**Figure 3:**
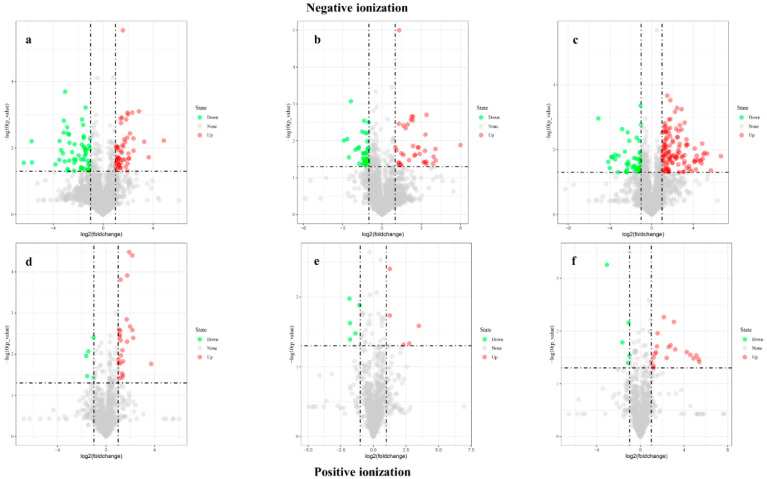
Analyze the volcano plots of genotype comparisons in both ionization modes. X > 1 and Y > 1.30 represent significant increases, while X < −1 and Y > 1.30 represent significant decreases. (**a**,**d**) A1 vs. X1, (**b**,**e**) B1 vs. Y1, (**c**,**f**) C1 vs. Z1. Red color indicates significant increases in metabolites and green indicates a significant decrease in metabolites.

Volcano plots was performed with selected metabolites based on VIP > 1.5, FC > 2.0, and *p* < 0.05. SB-DT2 showed increased upregulation of metabolites, followed by Matterhorn and SB-DT3. The number of downregulated metabolites was higher in Matterhorn than in SB-DT2. Compared to other genotypes, SB-DT3 (B1 vs. Y1) exhibited fewer upregulated and downregulated metabolites in both ionization modes (Figure 3).

### 3.6. Cluster Analysis

**Figure 4:**
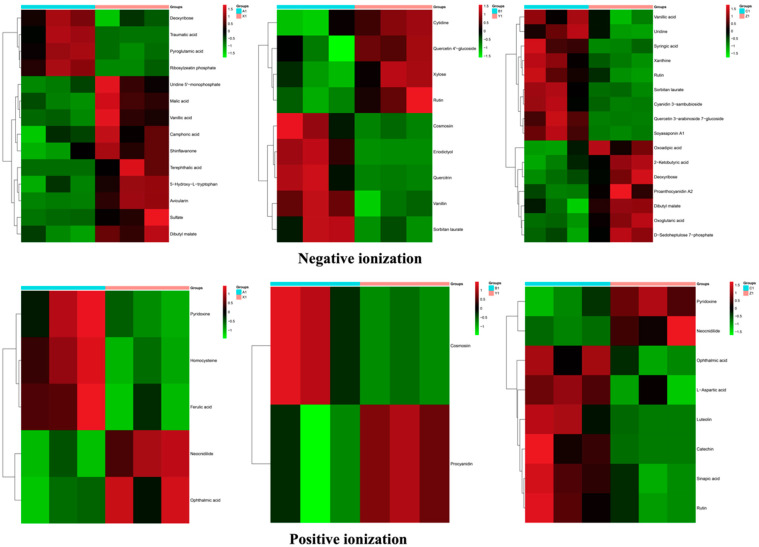
Hierarchical cluster analysis of metabolome data from the tolerant and sensitive genotypes in both ionization modes. SB-DT2 and stampede comparisons (C1 vs. Z1) show a higher accumulation of metabolites.

The abundance and correlation of metabolites in the tolerant and sensitive genotypes were analyzed with hierarchical cluster analysis (HCA). The metabolites with FC > 2, VIP > 1.5, and *p* < 0.05 were considered significant. More potential metabolites were identified in the SB-DT2 (C1 vs. Z1) than in other genotypes. Heatmap analysis revealed that ribosylzeatin phosphate, pyroglutamic acid, Deoxyribose, and traumatic acid were increased in Matterhorn (A1 vs. X1), deoxyribose, cosmosiin, eriodictyol, quercetin, vanillin, sorbitan laurate were enriched in SB-DT3 (B1 vs. Y1), vanillic acid, uridine, syringic acid xanthine, cyanidin 3-sambubioside, quercetin 3-arabinoside 7-glucoside, soyasaponin a pyridoxine, homocysteine, ferulic acid, ophthalmic acid, catechin, rutin, l-aspartic acid, sinapic acid and luteolin were accumulated in SB-DT2 (C1 vs. Z1). Dibutyl malate, deoxyribose, vanillic acid, sorbitan laurate, cosmosiin, rutin, pyridoxin, neocnidilide, and ophthalmic acid were common in all the comparisons in both ionization modes (Figure 4).

### 3.7. Identification of Potential Metabolites

Potential metabolites were selected with VIP > 1.5, log2 (fold change) > 1 or FC > 2, and *p* < 0.05 (Cai et al., 2020, Weljie et al., 2011, Zhang et al., 2019). We identified 28 potential metabolites in both ionization modes. Genotypes SB-DT2 (C1 vs. Z1) accumulated more metabolites. Metabolites such as ribosylzeatin phosphate, syringic acid, pyroglutamic acid, and deoxyribose were found in Matterhorn (A1 vs. X1), while SB-DT3 (B1 vs. Y1) contained quercetin, cosmosiin, eriodictyol, vanillin, sorbitan laurate metabolites. Cyanidin 3-sambubioside, quercetin 3-arabinoside 7-glucoside, sorbitan laurate, xanthine, soyasaponin A1, uridine, rutin, syringic acid, and vanillic acid were detected in SB-DT2 (C1 vs. Z1). All these metabolites were detected at negative ionization. The following metabolites were identified in each genotype using positive ionization. Homocysteine, c-pyridoxine, and ferulic acid were present in Matterhorn (A1 vs. X1). SB-DT3 contained only cosmosiin, while SB-DT2 contained catechin, luteolin, rutin, sinapic acid, and ophthalmic acid. We observed cosmosiin and rutin in both ionization modes. As a result, 26 were identified as possible potential metabolites (Table 1).

### 3.8. Dot Plot

**Figure 5:**
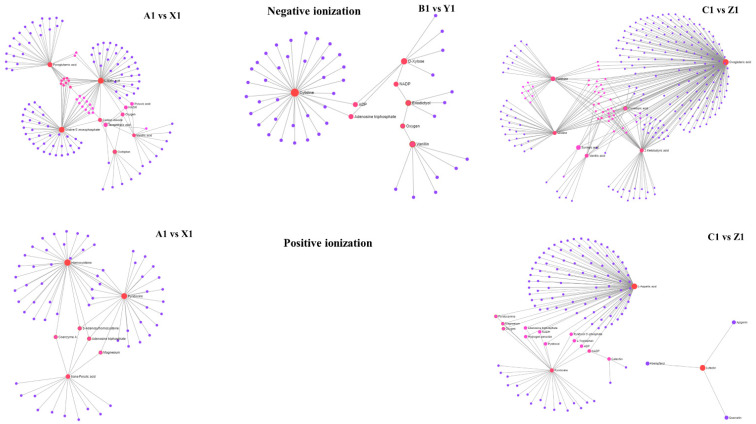
The metabolite–metabolite interaction network helps to highlight potential functional relationships between a wide set of annotated metabolites. Larger nodes with darker colors indicate the enriched metabolites are more important in the pathway. SB-DT2 (C1 vs. Z1) enriched with higher significant metabolites compared to other combinations.

The dot plot network illustrates the connection between the highly correlated metabolites with potential functional relationships. These metabolites were selected based on the MBRole (Metabolites Biological Role, Ibanez et al., 2016) with a *p* ≤ 0.05. As seen in Figure 5, the nodes with red color were enriched metabolites more important in the pathway. SB-DT2 contained more significant metabolites followed by Matterhorn and SB-DT3 (Table 2).

### 3.9. Correlation Network of Metabolites

**Figure 6:**
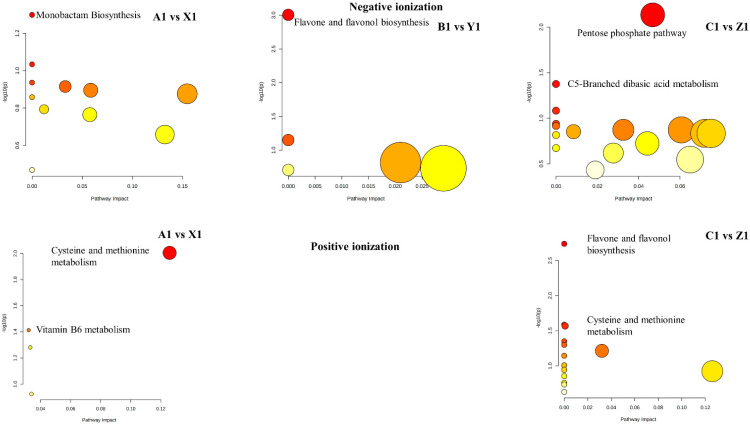
All matched pathways according to the *p* values from the pathway enrichment analysis and pathway impact values from the pathway topology analysis. The compound color within the pathway ranges from yellow to red, indicating different levels of significance of metabolites in the corresponding pathway. Circle size indicates the pathway impact score. SB-DT2 (C1 vs. Z1) enriched with more metabolic pathways than other genotypes. Corresponding pathways and their *p*-values are provided in Table S7.

The interrelationship among metabolites accumulated in the genotypes was analyzed by generating correlation networks. The correlation network diagram was constructed based on KEGG databases and MBRole. We obtained categorical annotations for pathways, enzyme interactions, and other biological processes from the significant metabolites. Monobactam biosynthesis, sulfur metabolism, pentose phosphate pathway, citrate cycle (TCA cycle), carbon fixation in photosynthetic organisms, tryptophan metabolism, glyoxylate and dicarboxylate metabolism, pyrimidine metabolism, purine metabolism, flavone and flavonol biosynthesis, flavonoid biosynthesis, arginine biosynthesis, alanine, aspartate and glutamate metabolism, glycine, serine, and threonine metabolism were metabolic pathways found frequently in both ionization modes (Table S6). SB-DT2 (C1 vs. Z1) has more enriched metabolic pathways, whereas SB-DT3 (B1 vs. Y1) results in fewer metabolic pathways (Figure 6). We also identified significant metabolic pathways with a *p* ≤ 0.05. These included monobactam biosynthesis and vitamin B6 metabolism in Matterhorn, cysteine and methionine metabolism (A1 vs. X1), flavone and flavonol biosynthesis in SB-DT3 (B1 vs. Y1), and pentose phosphate pathway, c5-branched dibasic acid metabolism, flavone and flavonol biosynthesis, cysteine and methionine metabolism, flavonoid biosynthesis and monobactam biosynthesis in SB-DT2 (C1 vs. Z1) (Table 3).

## 4. Discussion

A limited amount of research has been conducted on metabolomics studies related to the accumulation of seed metabolites in common bean plants under terminal drought stress [1]. This study compared three genotypes with different levels of drought tolerance to three sensitive genotypes in an attempt to understand the accumulation of genotype-specific metabolites in seeds under terminal drought conditions. Fold change of metabolites was deduced from comparisons between the tolerant and susceptible genotypes. The negative ionization mode observed more metabolite accumulation due to improved sensitivity and lower detection limits [1].

The accumulation of metabolites differs significantly by genotype, with specific metabolites increasing or decreasing based on genotypes [10]. There were significant increases in ribosylzeatin phosphate, pyroglutamic acid in Matterhorn, eriodictyol in SB-DT3, cyanidin 3-sambubioside, quercetin 3-arabinose 7-glucoside, and xanthine in SB-DT2. Each genotype likely has a different metabolic activity under drought stress [10,34]. SB-DT2 (C1 vs. Z1) had a higher accumulation of metabolites than other genotypes, demonstrating that genotype significantly influences metabolism [35]. Similarly, [36] (Guo et al., 2020) reported a higher accumulation of metabolites in the tolerant genotypes of wheat (Triticum aestivum) in response to drought stress. Moreover, metabolic response to drought stress is a dynamic and multifaceted process that depends on the strength, duration, and sensitivity of cultivars to stress [37].

Many of the accumulated metabolites play an influential role in drought stress. Pyroglutamic acid promotes drought tolerance by enhancing photosynthesis, antioxidant effects, and maintaining osmotic balance in lettuce (*Lactuca sativa*) plants [38]. Eriodictyol is a class of flavonoids (Flavanones). The content of eriodictyol in sorghum grains increased during water stress, and water stress and genotype interaction affected eriodictyol proportions and amounts [39]. Cyanidin 3-sambubioside and anthocyanin pigments protect from oxidative stress and metal toxicity in roselle [24]. Xanthine enhances drought tolerance in Arabidopsis [40]. In lentil (*Lens culinaris* Medik.), significant changes in xylose accumulation were observed during osmotic drought stress [41].

The abundance and correlation of potential metabolites were determined between the tolerant and sensitive genotypes. Ribosylzeatin phosphate, syringic acid, pyroglutamic acid, deoxyribose, homocysteine, pyridoxine, and ferulic acid were abundant in Matterhorn when compared to susceptible Sawtooth genotype. Similarly, quercetin, cosmosiin, eriodictyol, vanillin, and sorbitan laurate were increased in SB-DT3 compared to Merlot. The SB-DT2 genotype showed an increased accumulation of metabolites in comparison to Stampede. SB-DT2 is pinto germplasm developed through shuttle breeding and adapted to broad temperate and tropical conditions. Compared to SB-DT2, SB-DT3 has a lower geometric mean (GM), and its yield was reduced to 33.8% under drought stress [28]. We identified metabolites such as syringic acid, sorbitan laurate, cosmosiin, and rutin across genotypes. A substantial increase in syringic acid and rutin levels was observed in Amaranthus leafy vegetables during moderate and severe drought stress [42]. These compounds appear to contribute to a defense response associated with increased cell wall lignification in response to biotic and abiotic stresses [43].

Potential metabolites identified in the present study also play a crucial role in our health. Soyasaponins accumulated in SB-DT2 have strong adjuvant properties [44]. Cosmosiin is reported to have beneficial effects on diabetic complications by enhancing adiponectin secretion, tyrosine phosphorylation of insulin receptor-β, and GLUT4 translocation [45]. Syringic acid exhibits antioxidant, antimicrobial, anti-inflammatory, and antiendotoxic properties [46]. Additionally, syringic acid has been reported in Rosa *damascena* to protect from oxidative stress in water-limiting conditions [47].

There were differences in the metabolic pathways between genotypes based on individual comparisons. SB-DT2 and Matterhorn have more enriched metabolic pathways than SB-DT3. Monobactam biosynthesis was enriched for differentially expressed genes in climbing vine swallowworts (*Cynanchum auriculatum*) during salt stress and in foxtail millet (*Setaria italica*) under drought stress [48,49]. In the fruit body of Auricularia auricula (wood ear), the pentose phosphate pathway occurred as part of the survival response to drought stress [50]. Tryptophan metabolism was reported in chickpea (*Cicer arietinum*) in response to long term drought stress [51]. Abiotic and biotic stresses enhance flavonoid biosynthesis [52]. Flavonoids act as radical scavengers in Arabidopsis, protecting it from oxidative stress and drought [53].

## 5. Conclusions and Future Perspectives

The current study investigates the metabolic changes in common bean seeds of different genotypes under terminal drought stress. We compared tolerant and sensitive genotypes within the same race to better understand genotype-specific responses. Potential metabolite accumulation and related pathways were different in each comparison. Ribosylzeatin phosphate, syringic acid, pyroglutamic acid, deoxyribose in Matterhorn, quercetin, cosmosiin, eriodictyol, vanillin, sorbitan laurate in SB-DT3, and cyanidin 3-sambubioside, quercetin 3-arabinoside 7-glucoside, sorbitan laurate, xanthine, soyasaponin A1, uridine, rutin, syringic acid, vanillic acid in SB-DT2 were the potential metabolites accumulated under terminal drought stress. Several of these metabolites also have health benefits for humans besides alleviating drought stress. Possible metabolic pathways under terminal drought stress were identified in each comparison. Among them are monobactam biosynthesis, flavone and flavonol biosynthesis, pentose phosphate pathway, C5-branched dibasic acid metabolism, cysteine and methionine metabolism, flavonoid biosynthesis, and vitamin B6 metabolism with *p* < 0.05. These pathways are unique to each comparison. Overall, the SB-DT2 vs. Stampede comparisons showed enriched accumulation of potential metabolites and significant metabolic pathways. Thus, the present study will assist in understanding potential metabolites and their related pathways in the seeds of common bean tolerant genotypes in comparison to the sensitive genotypes under terminal drought stress. Further, this study will encourage transcriptomic and proteomic analyses to identify and understand the key metabolic genes and proteins in common bean seeds under drought stress. By conducting integrated studies, it becomes easier to understand the molecular mechanisms that govern drought stress responses, as well as changes in seed nutritional quality across genotypes.

## Figures and Tables

**Table 1:** Identification of potential metabolites in both ionization modes. These were selected with VIP > 1.5, log2 (fold change) > 1, and *p* < 0.05. A total of 26 metabolites was identified. Genotype SB-DT2 showed a higher accumulation of potential metabolites.

Negative Ionization			Positive Ionization		
A1 vs. X1 (Matterhorn)	B1 vs. Y1 (SB-DT3)	C1 vs. Z1 (SB-DT2)	A1 vs. X1 (Matterhorn)	B1 vs. Y1 (SB-DT3)	C1 vs. Z1 (SB-DT2)
Ribosylzeatin phosphate	Quercitrin	Cyanidin 3-sambubioside	Homocysteine	Cosmosiin	Catechin
	Cosmosiin	Quercetin 3-arabinoside 7-glucoside	Pyridoxine		Luteolin
Syringic acid	Eriodictyol	Sorbitan laurate	Ferulic acid		Rutin
Pyroglutamic acid	Vanillin	Xanthine			Sinapic acid
Deoxyribose	Sorbitan laurate	Soyasaponin A1			Ophthalmic acid
		Uridine			L-Aspartic acid
		Rutin			
		Syringic acid Vanillic acid			

**Table 2:** Significant metabolites identified in each genotype comparison shared the potential functional relationship. These are the enriched metabolites more important in pathways.

Negative Ionization			Positive Ionization	
A1 vs. X1	B1 vs. Y1	C1 vs. Z1	A1 vs. X1	C1 vs. Z1
Pyroglutamic acid	Cytidine	Oxoglutaric acid	Homocysteine	L-Aspartic acid
L-malic acid	Vanillin	2-Ketobutyric acid	Pyridoxine	Pyridoxine
Uridine 5′-monophosphate	Eriodictyol	Xanthine	Trans-Ferulic acid	Luteolin
Vanillic acid	D-Xylose	Uridine		
		Oxoadipic acid		
		Vanillic acid		
		Syringic acid		

**Table 3:** Significant metabolic pathway with *p* < 0.05 across the genotype comparisons.

Pathways	*p* < 0.05	Genotypes Comparisons
Monobactam biosynthesis	0.044826	Matterhorn (A1 vs. X1)
Flavone and flavonol biosynthesis	9.88 × 10^−4^	SB-DT3 (B1 vs. Y1), SB-DT2 (C1 vs. Z1)
Pentose phosphate pathway	0.0073327	SB-DT2 (C1 vs. Z1)
C5-Branched dibasic acid metabolism	0.042084	SB-DT2 (C1 vs. Z1)
Cysteine and methionine metabolism	0.0098777	Matterhorn (A1 vs. X1), SB-DT2 (C1 vs. Z1)
Vitamin B6 metabolism	0.038646	Matterhorn (A1 vs. X1)
Flavonoid biosynthesis	0.027047	SB-DT2 (C1 vs. Z1)

## Data Availability

Data is contained within the article or Supplementary Materials.

## Supplementary Materials

The following supporting information can be downloaded at: https://www.mdpi.com/article/10.3390/metabo12100944/s1, Figure S1: Genotypes comparisons. Figure S2: Flowchart of experimental design. Figure S3: Estimation of starch, protein contents and lipid percentage of genotypes. Mean values of three biological replicates with standard errors. Figure S4: Comparative accumulation of metabolites across genotypes with a fold change >2. Yellow indicates the common metabolites identified across genotypes. Green indicates the specific metabolites identified in each comparison. Figure S5: PLS-DA and OPLS-DA score plots of each comparison in both ionization modes showed clear separation of tolerant and sensitive genotypes for each comparison. Table S1: Listed below are the common bean genotypes and races grown in the field and subjected to terminal drought stress. Table S2: Mean yield (lbs/acre), days to flowering and maturity and 100 seed weight (SW) of the six genotypes evaluated near Scottsbluff, Nebraska, USA. Table S3: List of the metabolites obtained through negative and positive ionization modes provided in the table along with fold change and *p* values. Table S4: List of the metabolites with fold change >2. Across all genotype comparisons, negative ionization metabolites were found to be higher. Table S5: Variable importance in projection (VIP) > 1.5 was used in order to filter out metabolites significantly different between the tolerant and sensitive genotypes. In total, 79 metabolites were identified in negative ionization mode and 61 metabolites in positive ionization mode. The genotype SB-DT2 (C1 vs. Z1) contained more metabolites than the other genotypes. Table S6: Inferred metabolic pathway from accumulated metabolites using KEGG databases. There were more metabolic pathways associated with genotype SB-DT2. No pathways were found for SB-DT3 in the positive ionization mode.
