# Supplementary material for: Comparative Analysis of Untargeted Metabolomics in Tolerant and Sensitive Genotypes of Common Bean (Phaseolus vulgaris L.) Seeds Exposed to Terminal Drought Stress
Source: Metabolites. 2022 Oct 5;12(10):944. doi: 10.3390/metabo12100944 (PMC9607437; doi:10.3390/metabo12100944)

Figure S1 Genotypes comparisons

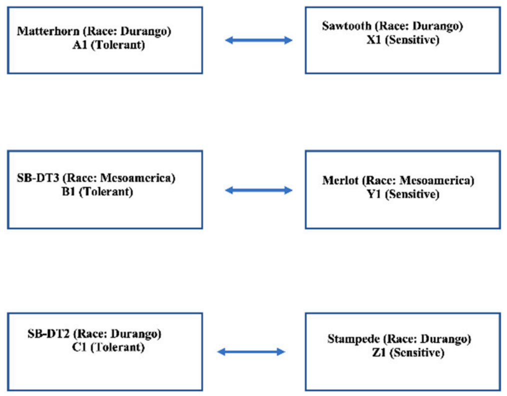

Figure S2 Flowchart of experimental design

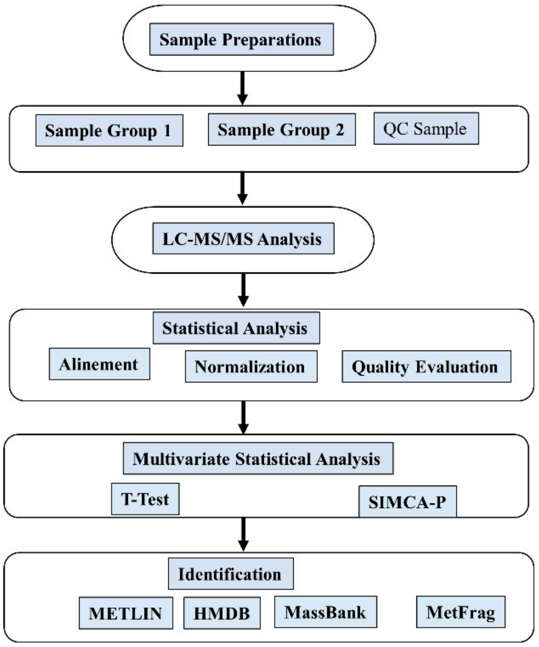

Figure S3 Estimation of starch, protein contents and lipid percentage of genotypes. Mean values of three biological replicates with standard errors

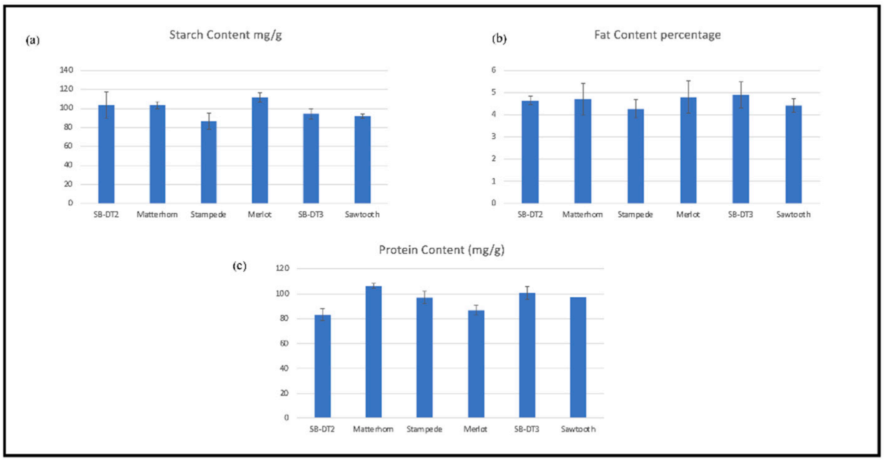

Figure S4 Comparative accumulation of metabolites across genotypes with a fold change > 2. Yellow indicates the common metabolites identified across genotypes. Green indicates the specific metabolites identified in each comparison

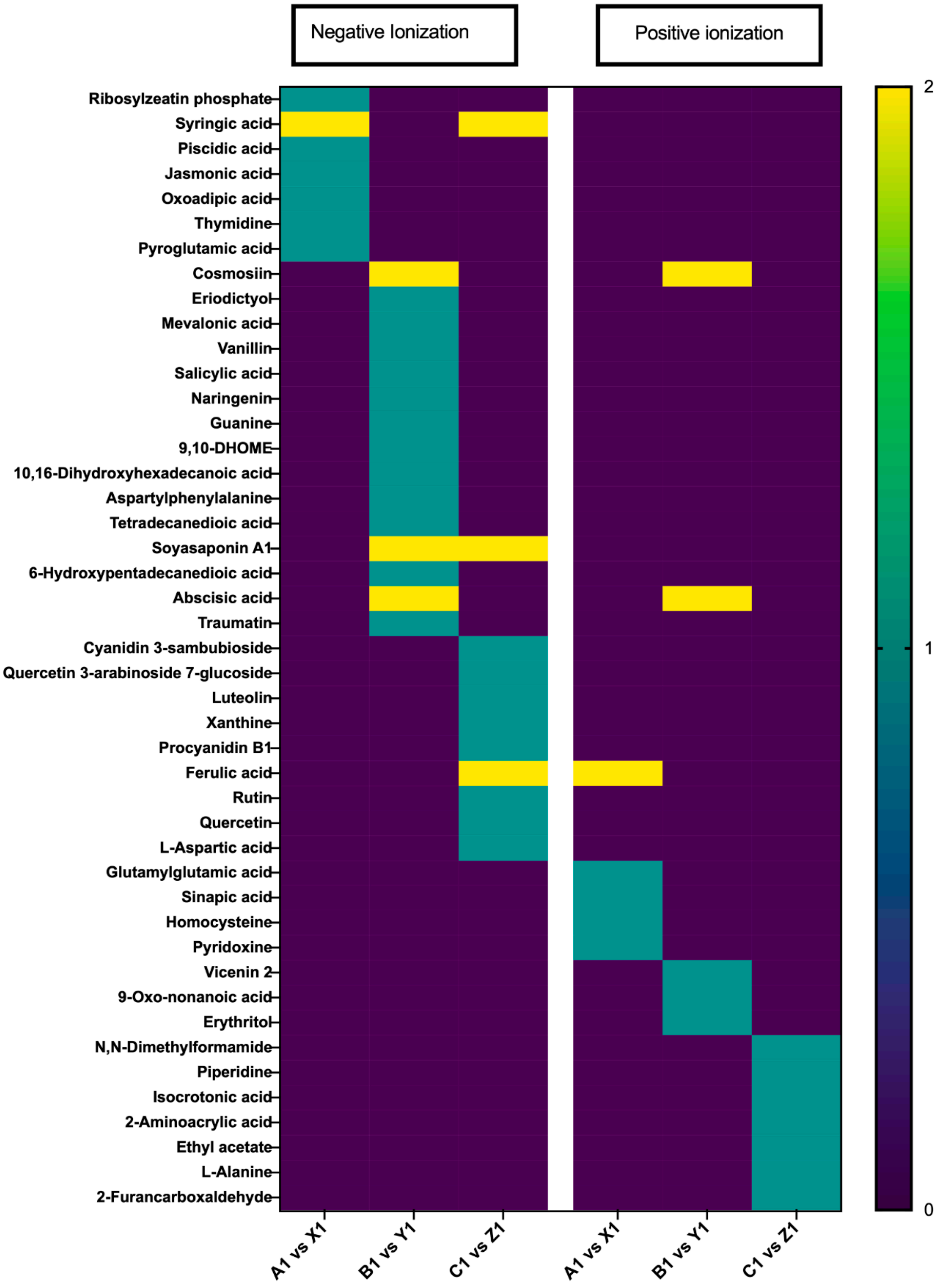

Figure S5 PLS-DA and OPLS-DA score plots of each comparison in both ionization modes showed clear separation of tolerant and sensitive genotypes for each comparison.

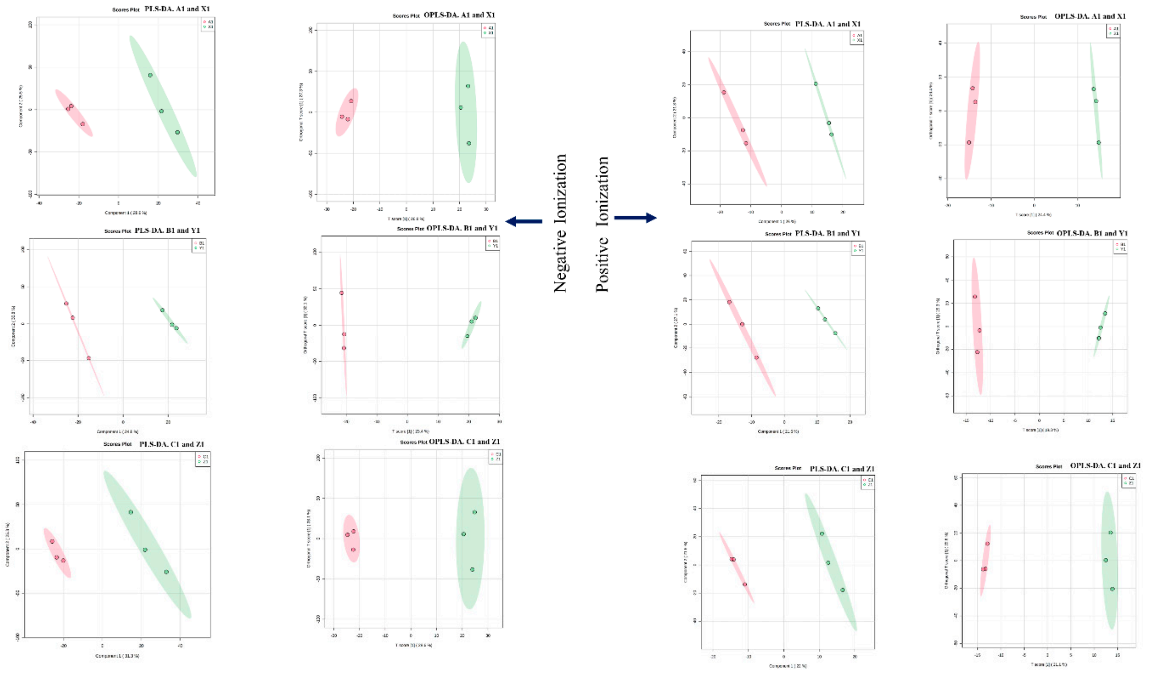

Supplement: Supplementary file 1 [file metabolites-12-00944-s001.zip › Figures 1-5.pdf]
