# Supplementary material for: Comparative Analysis of Untargeted Metabolomics in Tolerant and Sensitive Genotypes of Common Bean (Phaseolus vulgaris L.) Seeds Exposed to Terminal Drought Stress
Source: Metabolites. 2022 Oct 5;12(10):944. doi: 10.3390/metabo12100944 (PMC9607437; doi:10.3390/metabo12100944)
Supplement: Supplementary file 1 [file metabolites-12-00944-s001.zip › Table S1.pdf]

**Table S1**

Listed below are the common bean genotypes and races grown in the field and subjected to terminal drought stress.

| **S.NO** | **PEDIGREE** | | **Races** |
|---|---|---|---|
| 1 | Matterhorn | Tolerant | Durango |
| 2 | SB-DT2 | Tolerant | Durango |
| 3 | SB-DT3 | Tolerant | Mesoamerica |
| 4 | Stampede | Sensitive | Durango |
| 5 | Swatooth | Sensitive | Durango |
| 6 | Merlot | Sensitive | Mesoamerica |
