# Supplementary material for: Comparative Analysis of Untargeted Metabolomics in Tolerant and Sensitive Genotypes of Common Bean (Phaseolus vulgaris L.) Seeds Exposed to Terminal Drought Stress
Source: Metabolites. 2022 Oct 5;12(10):944. doi: 10.3390/metabo12100944 (PMC9607437; doi:10.3390/metabo12100944)
Supplement: Supplementary file 1 [file metabolites-12-00944-s001.zip › Table S2.pdf]

Table S2

Mean yield (lbs/acre), days to flowering and maturity and 100 seed weight (SW) of the six genotypes evaluated near Scottsbluff, Nebraska, USA.

**PEDIGREE**	**Yield lbs/acre**	**Flowering days**	**Maturity days**	**100-SW gr**
SB-DT2	2490	45	87	34.2
Matterhorn	2464	46	82	30.8
Stampede	2416	49	87	34.6
Merlot	2251	48	89	34.6
SB-DT3	1817	47	89	33.2
Sawtooth	1777	46	88	37.3
